# A study on the correlation of thyroid nodules with METS-IR and SII in a population undergoing health checkups

**DOI:** 10.3389/fendo.2025.1665432

**Published:** 2025-12-02

**Authors:** Ruoping Guan, Guokui Dai, Chuanjiang Ye, Xiangsheng Cai

**Affiliations:** 1 Clinical Laboratory, Guangzhou Cadre and Talent Health Management Center, Guangzhou, China; 2 Department of Ultrasound, Guangzhou Cadre and Talent Health Management Center, Guangzhou, China

**Keywords:** health checkup, thyroid nodules, METS-IR, SII, cross-sectional studies

## Abstract

**Objective:**

To investigate the associations between thyroid nodules and two emerging biomarkers-Metabolic Score for Insulin Resistance (METS-IR) and Systemic Immune-Inflammation Index (SII)-in adults undergoing routine health checkups.

**Methods:**

In this retrospective cross-sectional study, we analyzed data from 49,835 adults (65.50% male, 34.50% female) who underwent health checkups in 2023. Thyroid nodules were classified using the Thyroid Imaging Reporting and Data System (TI-RADS) categories (2, 3, ≥4). Statistical analyses, including chi-square tests and multiple logistic regression, were used to evaluate the relationships between nodule prevalence, sex, age, thyroid-stimulating hormone (TSH) levels, METS-IR, and SII.

**Results:**

Thyroid nodules were detected in 60.12% of the participants. The prevalence of TI-RADS 2, 3, and ≥4 nodules were 20.61%, 37.81%, and 1.69%, respectively. Nodule prevalence was significantly higher in women (70.07%) than in men (54.87%, P < 0.001). After multivariable adjustment, TI-RADS categories 2, 3, and ≥4 nodules were independently associated with female sex and increasing age (all P < 0.001). Notably, TI-RADS 2 and 3 nodules exhibited an inverse association with serum TSH levels (P < 0.001 for both), whereas TI-RADS 3 and ≥4 nodules showed positive associations with elevated METS-IR and SII values (P < 0.05 for all comparisons).

**Conclusion:**

Thyroid nodules are highly prevalent, particularly among women and older individuals. Lower-grade nodules (TI-RADS 2 and 3) show an inverse correlation with TSH levels, whereas higher-grade nodules (TI-RADS 3 and ≥4) are independently linked to increased insulin resistance (METS-IR) and systemic inflammation (SII). These findings suggest that METS-IR and SII could serve as valuable biomarkers for thyroid nodule assessment.

## Introduction

Thyroid nodules (TN) are a common endocrine system diagnosis during health checkups. Previous studies have demonstrated that the development of thyroid nodules is closely associated with factors such as age, body mass index (BMI), lipids, and blood glucose (1), with metabolic syndrome and insulin resistance identified as significant contributors to the rising incidence of thyroid nodules and papillary thyroid cancer (1, 2). Thyroid hormones influence glucose metabolism by acting on peripheral tissues, including the intestinal mucosa, liver, skeletal muscle, adipose tissue, and pancreas; progression of thyroid disease can ultimately lead to insulin resistance (3).

The Metabolic Score for Insulin Resistance (METS-IR) is a novel index for assessing insulin resistance and is linked to various chronic diseases. It has demonstrated superiority over the Homeostasis Model Assessment of Insulin Resistance (HOMA-IR) in predicting all-cause and cardiovascular mortality in the general population (4) and in forecasting the incidence of chronic kidney disease (CKD) (5). The Systemic Immune-Inflammation Index (SII) has emerged as a novel immune-inflammation index with broad applications (6, 7) and has been identified as an independent risk factor for thyroid nodules (8). Additionally, the benign or malignant nature of thyroid nodules has been linked to insulin resistance indices (HOMA-IR) (9) and *in vivo* immunoinflammatory factors (10). Ultrasonographic classification of thyroid nodules, based on the Thyroid Imaging Reporting and Data System (TI-RADS), categorizes nodules into five categories. However, no prior studies have examined the correlation between TI-RADS-classified thyroid nodules and METS-IR or SII. While most thyroid nodules show little change in imaging during follow-up, hematological indices may exhibit significant variations (11). To address this gap, the present study analyzed the relationship between different TI-RADS classifications of thyroid nodules and METS-IR and SII, evaluating their potential value in health checkups for thyroid nodules.

## Materials and methods

### Subjects

**Figure 1:**
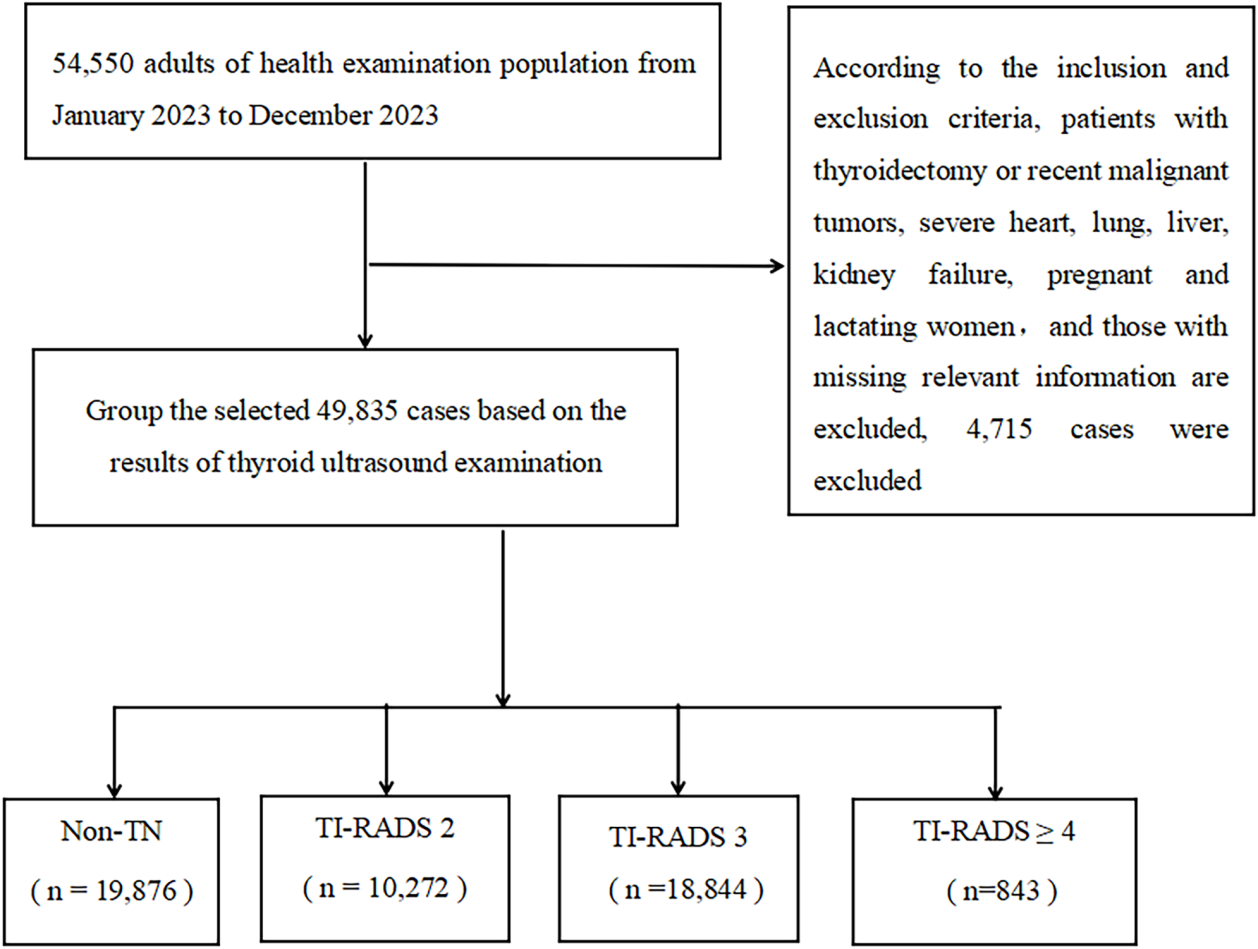
Flowchart for recruiting participants.

This retrospective cross-sectional study selected adults who underwent health checkups, including thyroid ultrasound and hematology tests, at the Guangzhou Cadre and Talent Health Management Center between January 2023 and December 2023. Inclusion criteria were: 1) age ≥18 years, and 2) complete data on thyroid ultrasound, BMI, blood pressure, and laboratory tests. Exclusion criteria included: 1) history of thyroidectomy or major surgery, 2) recent malignant tumors or severe cardiac, pulmonary, hepatic, or renal failure, and 3) pregnant or lactating women. A total of 49,835 subjects were enrolled, including 32,640 males (65.50%) and 17,195 females (34.50%), with ages ranging from 19 to 101 years (mean age: 48.45 ± 13.27 years). See **Figure 1**. All participants signed informed consent forms, and the study was approved by the Medical Ethics Committee of the Guangzhou Cadre and Talent Health Management Center (Approval No. K2022-10).

### Physical examination

Physical examinations were conducted by uniformly trained healthcare personnel. Height (m) and body mass (kg) were measured using calibrated height and weight scales, with BMI calculated as kg/m². Systolic blood pressure (SBP) and diastolic blood pressure (DBP) were measured using an OMRON (HBP-9020) automatic electronic sphygmomanometer. Participants rested quietly for 5–10 minutes before measurement, with the brachial artery of the right upper limb used for three measurements, the average of which was recorded (unit: mmHg).

### Echography

Thyroid ultrasound examinations were performed using a Siemens Acuson Sequoia color ultrasound diagnostic system with an 8–12 MHz probe frequency and thyroid-specific mode activated. Subjects were positioned supine with the head tilted backward to fully expose the neck. Thyroid ultrasound findings were categorized using the American College of Radiology Thyroid Imaging and Reporting Data Systems (ACR TI-RADS) grading system. Abnormal areas underwent repeated scanning to clarify lesion characteristics by observing ultrasound features such as nodule morphology, margins, echogenicity, and calcifications. Thyroid ultrasound evaluations were independently performed by two experienced attending physicians or higher-level practitioners. Nodule classifications were confirmed upon diagnostic agreement; discrepancies were resolved through consensus. Nodules were categorized according to the TI-RADS grading system as follows: No-RADS group, TI-RADS 2 group, TI-RADS 3 group, and TI-RADS ≥4 group.

### Laboratory examination

Fasting venous blood was collected from subjects, and serum was separated to measure 25-hydroxyvitamin D (25(OH)D) using a Roche cobas e 801 electrochemiluminescence meter. Fasting blood glucose (FPG), triacylglycerol (TG), total cholesterol (TCHO), high-density lipoprotein cholesterol (HDL-C), low-density lipoprotein cholesterol (LDL-C), urea, blood creatinine (Scr), and blood uric acid (BUA) were analyzed using a Canon TBA FX-8 biochemistry meter. The estimated glomerular filtration rate (eGFR) was calculated using the CKD-EPI 2021 formula (12). Two milliliters of EDTA-K2 anticoagulant whole blood were collected, and blood cell counts were performed using Mindray BC-6800 Plus hematology analyzer. The following indices were calculated:


$$\begin{array}{l} \begin{array}{l} \text{NLR }=\text{ neutrophil count }/\text{ lymphocyte count,}\\ \text{PLR }=\text{ platelet count }/\text{ lymphocyte count,}\\ \text{MHR }=\text{ monocyte count }/\text{ HDL}-\text{C,}\\ \text{SII }=\;\left(\text{neutrophil count }\times \text{ platelet count}\right)\;/\text{ lymphocyte count}, \end{array}\\ \text{METS}-\text{IR}=\text{ln }\left[2\;\times \text{ FPG }\left(\text{mg}/\text{dL}\right)+\text{TG }\left(\text{mg}/\text{dL}\right)\right]\;\times \text{ BMI}/\text{ln }\left[\text{HDL}-\text{C }\left(\text{mg}/\text{dl}\right)\right]. \end{array}$$

### Statistical processing

Data analysis was performed using SPSS 25.0 software. Non-normally distributed quantitative data are expressed as mean (interquartile range). Measurement data were analyzed using t-tests or rank-sum tests (Kruskal-Wallis test), respectively. Count data were expressed as frequencies and proportions and analyzed using chi-square tests. Multivariate logistic regression analysis was employed to identify risk factors associated with TI-RADS 2, 3, and ≥4 thyroid nodules and to assess their association with each factor. The significance level was set at α= 0.05, with P < 0.05 indicating statistical significance.

## Results

### Clinical data of the study population

**Figure 2:**
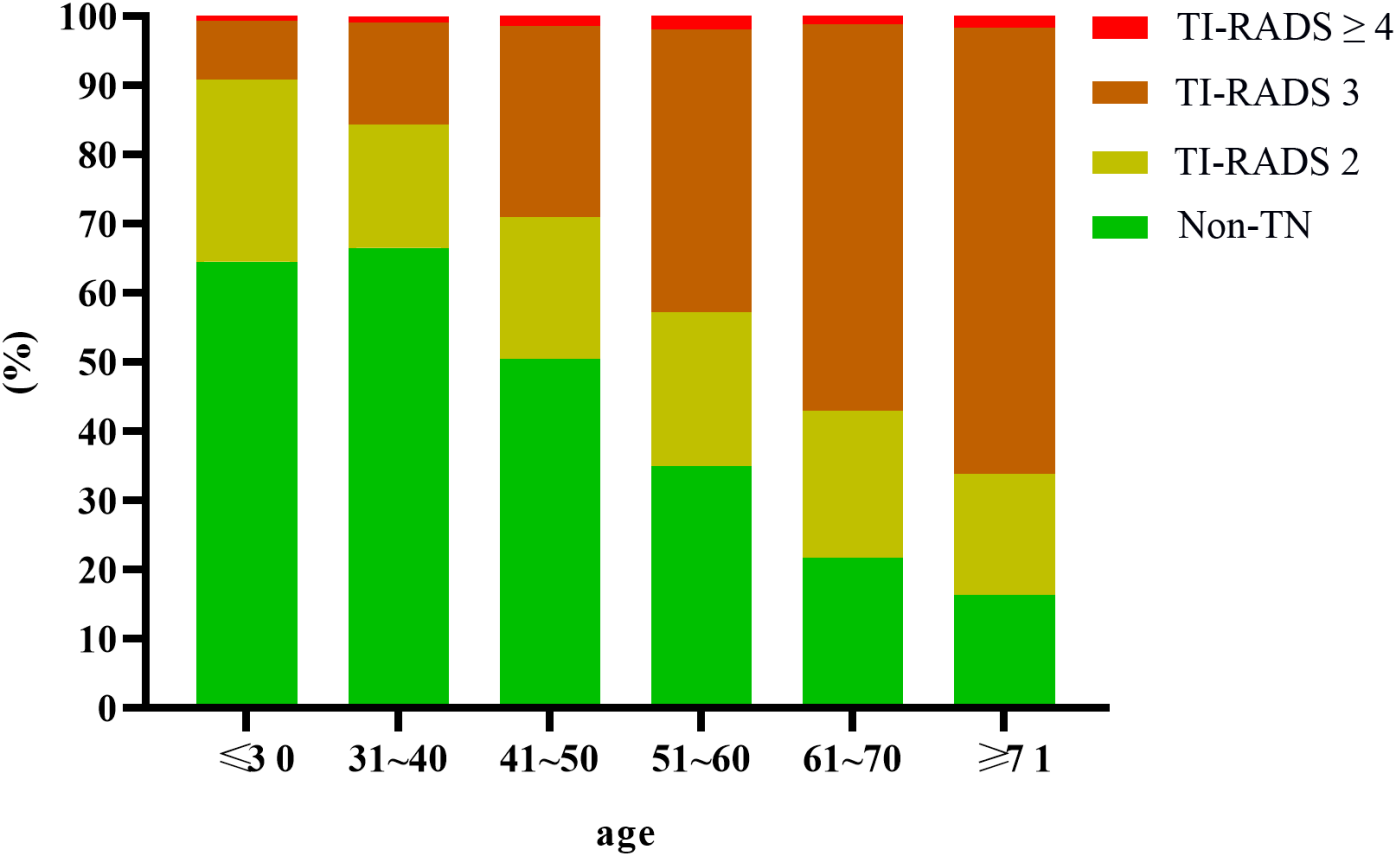
Distribution of TN in adult males.

**Figure 3:**
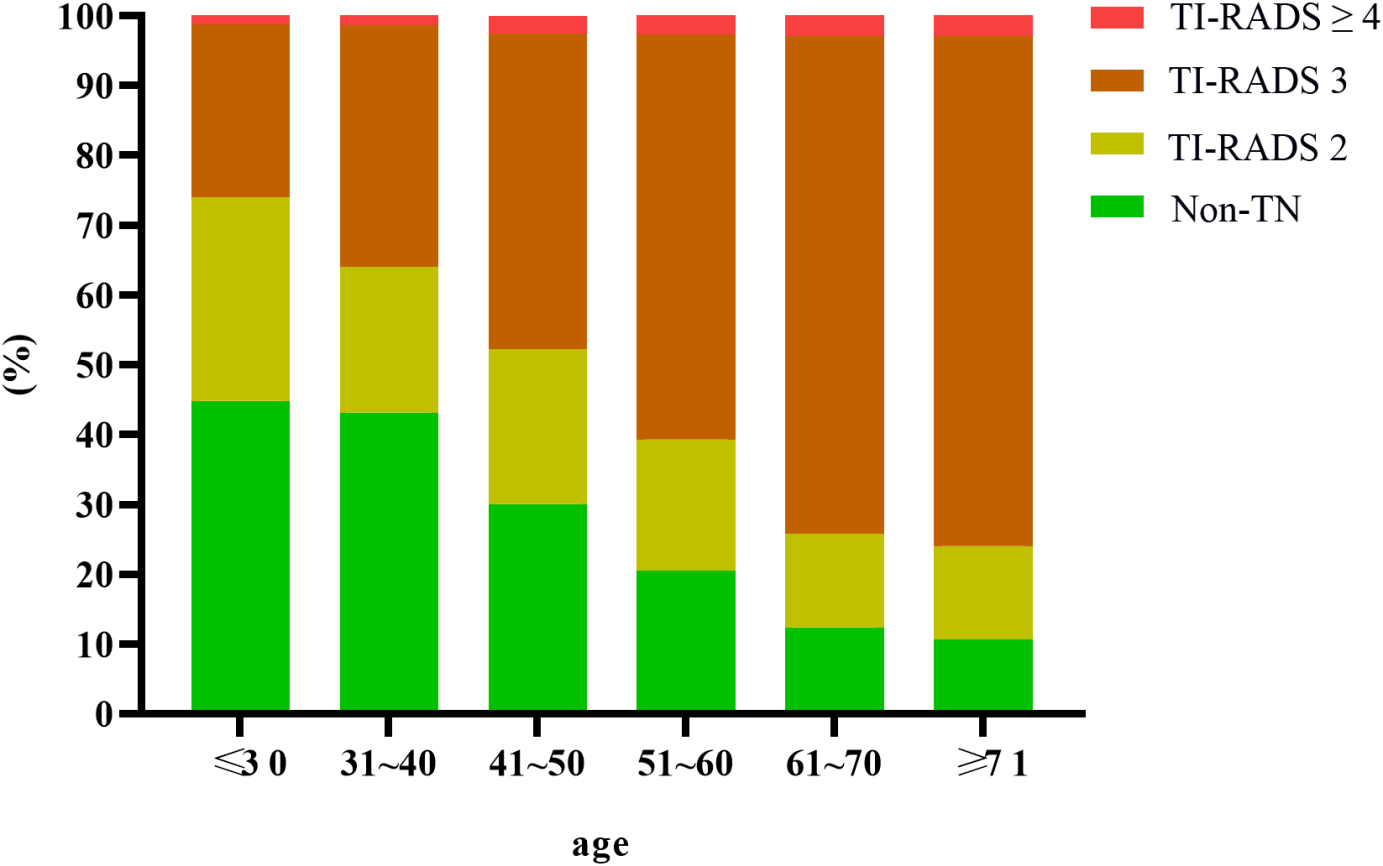
Distribution of TN in adult females.

Among 49,835 subjects, 29,959 cases of thyroid nodules were detected by ultrasound, yielding a detection rate of 60.12%. The detection rates for TI-RADS 2, 3, and ≥4 nodules were 20.61% (10,272/49,835), 37.81% (18,844/49,835), and 1.69% (843/49,835), respectively. The detection rate was significantly higher in females (70.07%, 12,048/17,195) than in males (54.87%, 17,911/32,640), with a statistically significant difference (P<0.001). See **Table 1**, **Figures 2**, **3**.

**Table 1:** The distribution of thyroid nodule [ n ( % ) ].

Age (years)	Male					Female				
	n	Non-TN	TI-RADS 2	TI-RADS 3	TI-RADS≥4	n	Non-TN	TI-RADS 2	TI-RADS 3	TI-RADS ≥4
≤ 30	2248	1450 (64.50)	592 (26.33)	191 (8.50)	15 (0.67)	1894	849 (44.83)	552(29.14)	468 (24.71)	25 (1.32)
31 ~ 40	6257	4163 (66.53)	1115 (17.82)	917 (14.66)	62 (0.99)	4546	1963 (43.18)	948(20.85)	1572 (34.58)	63 (1.39)
41 ~ 50	9812	4950 (50.45)	2012 (20.50)	2710 (27.62)	140 (1.43)	4229	1271 (30.06)	940(22.23)	1910 (45.16)	108 (2.55)
51 ~ 60	8876	3098 (34.90)	1981 (22.32)	3624 (40.83)	173 (1.95)	3402	697 (20.49)	641(18.84)	1974 (58.02)	90 (2.65)
61 ~ 70	3284	714 (21.74)	695 (21.16)	1837 (55.94)	38 ( 1.16 )	1959	243 (12.41)	262(13.37)	1396 (71.26)	58 (2.96)
≥ 71	2163	354 (16.37)	378 (17.48)	1394 (64.45)	37 (1.70)	1165	124 (10.64)	156(13.39)	851 (73.05)	34 (2.92)

Compared with participants without thyroid nodules, those with TI-RADS 2, 3, or ≥4 nodules differed significantly in multiple demographic, metabolic, and inflammatory parameters, including sex distribution, age, BMI, systolic and diastolic blood pressure, 25(OH)D, TSH, FT3, FT4, fasting glucose, triglycerides, total cholesterol, HDL-C, eGFR, uric acid, NLR, PLR, SII, MHR, and METS-IR (all P < 0.05). No significant difference was found in LDL-C levels between groups (P > 0.05). See **Table 2**.

**Table 2:** Clinical data of the study population [ n ( % ), $\bar{x}\pm s$ ].

	Non-TN	TI-RADS 2	TI-RADS 3	TI-RADS ≥4	F / χ*2*	*P*
Total	19876 (39.88)	10272 (20.61)	18844 (37.81)	843 (1.69)	/	/
Male	14729 (45.13)	6773 (20.75)	10673 (32.70)	465 (1.42)	**χ2 =** 461.322	**< 0.001**
Female	5147 (29.93)	3499 (20.35)	8171 (47.52)	378 (2.20)		
Age (years)	43.79 ± 11.33	47.33 ± 13.04	53.80 ± 13.32	51.35 ± 12.69	F = 2111.008	**< 0.001**
BMI (kg/m^2^)	24.20 ± 3.22	24.15 ± 3.17	24.29 ± 3.24	24.55 ± 3.37	F = 7.712	**< 0.001**
SBP (mmHg)	120.4 ± 14.9	121.6 ± 15.6	125.3 ± 17.2	125.7 ± 17.6	F = 330.108	**< 0.001**
DBP (mmHg)	72.3 ± 10.8	72.3 ± 10.8	73.4 ± 11.1	73.8 ± 11.2	F = 45.244	**< 0.001**
25 (OH) D(ng/mL)	22.43 ± 6.75	22.78 ± 6.78	22.86 ± 7.20	22.39 ± 6.87	F = 3.000	**0.029**
TSH (Q1, Q3) uIU / mL)	1.97 ( 1.38,2.83 )	1.87 ( 1.33,2.69 )	1.81 ( 1.26,2.64 )	1.91 ( 1.29,2.71 )	F = 13.072	**< 0.001**
FT3 (ng/mL)	4.99 ± 1.30	4.88 ± 0.77	4.87 ± 1.05	4.77 ± 0.61	F = 25.188	**< 0.001**
FT4 (ng/mL)	16.97 ± 3.58	16.82 ± 2.58	16.69 ± 3.18	16.83 ± 2.47	F = 7.711	**< 0.001**
FPG (mmol/L)	5.23 ± 0.96	5.34 ± 1.12	5.50 ± 1.26	5.51 ± 1.39	F = 196.838	**< 0.001**
TG [ M (Q1, Q3), mmol / L]	1.19 ( 0.84,1.78 )	1.18 ( 0.83,1.74 )	1.21 ( 0.86,1.78 )	1.25 ( 0.87,1.89 )	F = 6.325	**< 0.001**
TCHO (mmol/L)	5.16 ± 0.96	5.18 ± 0.99	5.21 ± 1.02	5.24 ± 1.03	F = 11.357	**< 0.001**
HDL-C (mmol/L)	1.47 ± 0.34	1.50 ± 0.36	1.51 ± 0.37	1.50 ± 0.37	F = 52.997	**< 0.001**
LDL-C (mmol/L)	2.97 ± 0.82	2.97 ± 0.83	2.97 ± 0.86	2.98 ± 0.86	F = 0.189	0.904
eGFR (min·1.73 m^2^)	101.0 ± 11.3	99.2 ± 16.0	95.4 ± 16.2	97.0 ± 16.0	F = 432.210	**< 0.001**
BUA (umol/L)	411.9 ± 102.5	400.0 ± 102.0	391.7 ± 101.3	396.6 ± 101.9	F = 128.622	**< 0.001**
METS-IR	31.81 ± 6.12	30.60 ± 6.00	31.89 ± 6.10	32.43 ± 6.37	F = 8.091	**< 0.001**
NLR	1.82 ± 0.81	1.83 ± 0.82	1.88 ± 0.81	1.94 ± 0.95	F = 19.478	**< 0.001**
PLR	128.8 ± 43.6	129.7 ± 46.9	130.0 ± 47.2	136.0 ± 48.3	F = 8.234	**< 0.001**
SII	450.3 ± 251.2	456.9 ± 244.3	469.6 ± 250.0	499.5 ±2 99.6	F = 26.581	**< 0.001**
MHR	0.28 ± 0.13	0.28 ± 0.13	0.28 ± 0.13	0.2 8± 0.13	F = 3.954	**0.008**

Bold P values indicate statistical significance at P < 0.05.

### Analysis of exposure factors for thyroid nodules

After adjusting for variables such as gender, age, and blood pressure, a multiple logistic regression analysis was conducted with the presence of thyroid nodules as the dependent variable and TSH, SII, and METS-IR as independent variables. Results showed that all thyroid nodules were independently associated with female gender and increasing age (all P < 0.001). TI-RADS 2 and 3 nodules showed independent negative correlations with TSH levels (TI-RADS 2: OR = 0.956, 95% CI: 0.936–0.977, P < 0.001; TI-RADS 3: OR = 0.923, 95% CI: 0.904–0.943, P < 0.001). TI-RADS 3 and ≥4 nodules were positively correlated with METS-IR (TI-RADS 3: OR = 1.019, 95% CI: 1.011–1.026, P <0.001; TI-RADS ≥4: OR = 1.024, 95% CI: 1.004–1.044, P = 0.019). TI-RADS 3 and ≥4 nodules were positively correlated with SII (TI-RADS 3: OR = 1.004, 95% CI: 1.002–1.005, P < 0.001; TI-RADS ≥4: OR = 1.005, 95% CI: 1.001–1.009, P = 0.020). See **Table 3**.

**Table 3:** Logistic regression analysis of TN groups.

Variables	β	S.E.	Wald χ^2^	p value	OR(95%CI)
TI-RADS 2 group compare with control group					
Female	0.448	0.053	71.455	**<0.001**	1.565(1.411,1.736)
Age	0.025	0.002	151.635	**<0.001**	1.025(1.021,1.029)
TSH	-0.045	0.011	16.234	**<0.001**	0.956(0.936,0.977)
METS-IR	0.002	0.004	0.131	0.718	1.002(0.993,1.010)
SII/10	0.001	0.001	2.056	0.152	1.001(0.900,1.003)
TI-RADS 3 group compare with control group					
Female	1.097	0.046	574.465	**<0.001**	2.994(2.737,3.275)
Age	0.064	0.002	1357.298	**<0.001**	1.066(1.062,1.070)
TSH	-0.080	0.011	55.214	**<0.001**	0.923(0.904,0.943)
METS-IR	0.018	0.004	24.391	**<0.001**	1.019(1.011,1.026)
SII/10	0.004	0.001	16.811	**<0.001**	1.004(1.002,1.005)
TI-RADS 4~ group compare with control group					
Female	1.018	0.124	67.503	**<0.001**	2.769(2.171,3.530)
Age	0.054	0.004	153.127	**<0.001**	1.056(1.047,1.065)
TSH	-0.025	0.019	1.799	0.180	0.975(0.939,1.012)
METS-IR	0.024	0.010	5.462	**0.019**	1.024(1.004,1.044)
SII/10	0.005	0.002	5.422	**0.020**	1.005(1.001,1.009)

The comparison group is Non-TN group, and the SII value is divided by 10 and included in the analysis.

Bold P values indicate statistical significance at P < 0.05.

### Subgroup analysis and interaction analysis

Further subgroup analysis was conducted based on gender and age (≤45 years as the younger group, >45 years as the older group). Results showed that in the female and older subgroups, TI-RADS ≥4 nodules was negatively correlated with TSH levels (OR = 1.150, 95%CI: 1.040–1.260, P = 0.006; OR = 1.210, 95%CI: 1.100–1.340, P <0.001). Among TI-RADS 2 nodules, only the young subgroup showed a positive correlation with METS-IR (OR = 1.010, CI: 1.000–1.010, P = 0.01). No other TI-RADS 2 subgroups demonstrated an association with METS-IR. All TI-RADS 3 subgroups were positively correlated with METS-IR (all P < 0.01). Both male and female subgroups, as well as the young subgroup, in TI-RADS ≥4 categories showed positive correlations with METS-IR (all P < 0.001). The older subgroup in TI-RADS ≥4 categories showed no correlation with METS-IR (P = 0.220). The correlations between thyroid nodule subgroups and SII were consistent with the logistic regression results. See **Table 4**.

**Table 4:** Association of TSH, METS-IR, and SII (per 10-unit increase) with thyroid nodule risk across TI-RADS categories, stratified by sex and age.

Subgroup	Exposure	TI–RADS 2		TI–RADS 3		TI–RADS ≥4	
		OR (95% CI)	P value	OR (95% CI)	P value	OR (95% CI)	P value
TSH_rev	Male	1.050 (1.020–1.090)	**0.004**	1.100 (1.060–1.140)	**<0.001**	1.000 (0.970–1.030)	0.850
	Female	1.080 (1.040–1.120)	**<0.001**	1.060 (1.030–1.080)	**<0.001**	1.150 (1.040–1.260)	**0.006**
	≤45 y	1.110 (1.060–1.160)	**<0.001**	1.070 (1.030–1.110)	**<0.001**	0.990 (0.970–1.010)	0.180
	>45 y	1.050 (1.020–1.080)	**0.001**	1.070 (1.050–1.100)	**<0.001**	1.210 (1.100–1.340)	**<0.001**
METS-IR	Male	1.000 (0.997–1.010)	0.330	1.020 (1.010–1.020)	**<0.001**	1.040 (1.020–1.050)	**<0.001**
	Female	1.000 (0.996–1.010)	0.320	1.020 (1.020–1.030)	**<0.001**	1.040 (1.020–1.060)	**<0.001**
	≤45 y	1.010 (1.000–1.010)	**0.010**	1.040 (1.030–1.040)	**<0.001**	1.080 (1.070–1.100)	**<0.001**
	>45 y	1.000 (0.992–1.000)	0.480	1.010 (1.000–1.010)	**0.001**	1.010 (0.994–1.030)	0.220
SII_10	Male	1.000 (0.999–1.000)	0.620	1.003 (1.002–1.004)	**<0.001**	1.004 (1.001–1.006)	**0.006**
	Female	1.000 (0.999–1.000)	0.220	1.003 (1.002–1.004)	**<0.001**	1.006 (1.003–1.010)	**<0.001**
	≤45 y	1.000 (1.000–1.000)	0.150	1.003 (1.002–1.005)	**<0.001**	1.007 (1.004–1.010)	**<0.001**
	>45 y	1.000 (0.999–1.000)	0.850	1.003 (1.001–1.004)	**<0.001**	1.003 (1.001–1.006)	**0.008**

TSH_rev: reverse-coded thyroid-stimulating hormone (higher values indicate lower TSH); METS-IR: metabolic score for insulin resistance; SII_10: systemic immune-inflammation index scaled per 10 units. ORs are reported with 95% confidence intervals in parentheses. P values < 0.001 are reported as "<0.001" for clarity and space efficiency. Age groups: ≤45 years vs. >45 years.

Bold P values indicate statistical significance at P < 0.05.

### Interaction between TSH, METS-IR, and SII in thyroid nodules

TSH in TI-RADS ≥4 thyroid nodules interacted with gender (P = 0.009) and older age (P < 0.001). METS-IR in TI-RADS 3 and ≥4 thyroid nodules interacted with older age (both P < 0.001). See **Table 5**.

**Table 5:** Interaction effects between exposures and demographic factors across TI-RADS categories.

TI-RADS	Exposure	Stratification Variable	Interaction Term	OR (95% CI)	P for Interaction
Category 2	TSH_rev	Sex (Ref: Male)	TSH_rev × Female	1.030 (0.980–1.090)	0.200
	TSH_rev	Age >45 (Ref: ≤45)	TSH_rev × Age >45	0.950 (0.900–1.010)	0.081
	METS-IR	Sex (Ref: Male)	METS-IR × Female	1.000 (0.990–1.010)	0.780
	METS-IR	Age >45 (Ref: ≤45)	METS-IR × Age >45	1.000 (0.990–1.010)	0.460
	SII_10	Sex (Ref: Male)	SII_10 × Female	1.000 (1.000–1.000)	0.340
	SII_10	Age >45 (Ref: ≤45)	SII_10 × Age >45	1.000 (0.997–1.001)	0.230
Category 3	TSH_rev	Sex (Ref: Male)	TSH_rev × Female	0.970 (0.930–1.010)	0.120
	TSH_rev	Age >45 (Ref: ≤45)	TSH_rev × Age >45	1.010 (0.970–1.060)	0.640
	METS-IR	Sex (Ref: Male)	METS-IR × Female	1.000 (0.990–1.010)	0.410
	METS-IR	Age >45 (Ref: ≤45)	METS-IR × Age >45	0.980 (0.980–0.990)	**<0.001**
	SII_10	Sex (Ref: Male)	SII_10 × Female	1.000 (1.000–1.000)	0.650
	SII_10	Age >45 (Ref: ≤45)	SII_10 × Age >45	1.000 (0.997–1.001)	0.360
Category 4 and above	TSH_rev	Sex (Ref: Male)	TSH_rev × Female	1.140 (1.030–1.260)	**0.009**
	TSH_rev	Age >45 (Ref: ≤45)	TSH_rev × Age >45	1.220 (1.110–1.350)	**<0.001**
	METS-IR	Sex (Ref: Male)	METS-IR × Female	1.010 (0.980–1.030)	0.680
	METS-IR	Age >45 (Ref: ≤45)	METS-IR × Age >45	0.940 (0.920–0.960)	**<0.001**
	SII_10	Sex (Ref: Male)	SII_10 × Female	1.000 (1.000–1.010)	0.350
	SII_10	Age >45 (Ref: ≤45)	SII_10 × Age >45	0.997 (0.993–1.001)	0.150

ORs are presented with 95% confidence intervals in parenthesesP values < 0.001 are reported as "<0.001".Reference categories: Sex = Male; Age group = ≤45 years.TSH_rev: reverse-coded thyroid-stimulating hormone; METS_IR: metabolic score for insulin resistance; SII_10: systemic immune-inflammation index (scaled by 10).

Bold P values indicate statistical significance at P < 0.05.

## Discussion

The global prevalence of thyroid nodules has been increasing, a trend that cannot be attributed solely to advancements in diagnostic technology (11). In this study, conducted among 49,835 individuals undergoing health checkups, the detection rate of thyroid nodules was 60.12%, with a significantly higher prevalence in women (70.07%) compared to men (54.87%, P < 0.001). This finding aligns with some studies (13) but exceeds others (14, 15), likely due to variations in age, gender distribution, geographic location, lifestyle, environmental factors, and iodine intake levels (16–18). For instance, both iodine deficiency and excess can influence thyroid nodule occurrence (16). Age emerges as a key risk factor, with thyroid nodule prevalence exceeding 75% among individuals aged 60 and above in Germany (19). Findings from this study indicate that for each additional year of age, the risk of TI-RADS 2 to ≥4 nodules increases by 2.5% to 6.6%, with the strongest correlation observed between TI-RADS 3 nodules and age. Age emerged as a critical risk factor, with the risk of TI-RADS 2 to ≥4 nodules increasing by 2.5% to 6.6% per year of age, with TI-RADS 3 nodules showing the strongest age-related association. Women exhibited a 1.565 to 2.994 times higher risk of developing all types of thyroid nodules compared to men, potentially due to the thyroid-growth-promoting effects of estrogen and progesterone (1, 17, 18). Estrogen may regulate thyroid cell proliferation and differentiation, increasing nodule formation risk (18).

Thyroid-stimulating hormone (TSH) serves as a fundamental indicator for assessing thyroid function and plays a pivotal role in thyroid nodule formation. As a crucial growth factor for thyroid cells, abnormal TSH levels—whether elevated or suppressed—constitute risk factors for thyroid nodules (20). TSH not only promotes nucleic acid and protein synthesis in thyroid follicular epithelial cells but also stimulates thyroid cell proliferation, thereby increasing glandular size. Abnormal adult TSH levels correlate with increased risks of goiter and antibody positivity. Even among adults with normal TSH levels, relatively low TSH levels constitute a risk factor for TN (18, 20). This study found that both TI-RADS Grade 2 and Grade 3 nodules exhibited a negative correlation with TSH levels (P < 0.001). This inverse relationship may reflect TSH suppression due to increased thyroid hormone secretion or be associated with subclinical hyperthyroidism linked to low-grade nodules. Low TSH levels may influence nodule formation by altering proliferative signaling pathways, such as the TSH receptor signaling pathway (18). Further research is needed to elucidate the mechanism of TSH action across different TI-RADS classifications.

A key innovation of this study lies in its first-ever reporting of associations between thyroid nodules classified under different TI-RADS categories and the Metabolic Score for Insulin Resistance (METS-IR) as well as the Systemic Inflammation Index (SII). METS-IR is calculated based on conventional clinical indicators (fasting blood glucose, triglycerides, HDL-C, and BMI) and demonstrates superior predictive performance compared to HOMA-IR for cardiovascular disease, all-cause mortality (4), and chronic kidney disease incidence (5). This study found a positive correlation between METS-IR levels and the young subgroup of TI-RADS 2-grade TN (P < 0.05). TI-RADS grade 3 and ≥4 TN showed a positive correlation with higher METS-IR levels (P < 0.05), suggesting insulin resistance may play a significant role in the development of high-risk nodules. A bidirectional association may exist between thyroid hormones and insulin resistance, as insulin resistance can cause thyroid dysfunction, while altered thyroid function may increase circulating lipid levels and exacerbate insulin resistance (21). Components of metabolic syndrome, such as obesity and dyslipidemia, may further amplify nodule risk by influencing thyroid hormone metabolism and inflammatory pathways (2, 22). Insulin resistance-induced hyperinsulinemia may stimulate thyroid cell proliferation via insulin-like growth factor-1 (IGF-1) receptors (23), with elevated insulin exerting stimulatory effects on cellular replication and anti-apoptotic actions, thereby promoting tissue hyperplasia and proliferation (24) that facilitate nodule formation. Concurrently, insulin resistance may even promote papillary thyroid carcinoma (PTC) development through inflammatory pathways and cellular proliferation (25). Metabolic syndrome-related insulin resistance (METS-IR) shows a positive correlation with medullary thyroid carcinoma incidence (26). This suggests a potential approach for preventing and managing thyroid nodules and related diseases: could controlling metabolic indicators to reduce insulin resistance levels effectively lower the occurrence and progression of endocrine disorders, including thyroid conditions?

The Systemic Immune-Inflammation Index (SII), reflecting systemic immune-inflammatory status, is increasingly applied in diseases like cancer and cardiovascular conditions (6, 7). This study found that TI-RADS 3 and ≥4 nodules were independently associated with higher SII levels (*P* < 0.05), reinforcing prior findings that SII is an independent risk factor for thyroid nodules (8). However, SII demonstrates limited predictive capability for thyroid nodules (8). This may be attributed to the complex etiology of thyroid nodules, as well as the absence of graded studies in populations with thyroid nodules. Chronic inflammation may drive thyroid cell proliferation and tumor formation through pro-inflammatory cytokines (e.g., TNF-α, IL-6) and vascular endothelial growth factor (VEGF) (25). The inflammatory microenvironment may also increase the malignant potential of thyroid nodules via oxidative stress and DNA damage (27). These SII findings underscore the role of systemic inflammation in higher-grade nodule pathophysiology.

Compared to prior studies, this research offers several novel contributions. While previous studies focused on thyroid nodules and metabolic syndrome or HOMA-IR (9, 10), this is the first to use METS-IR with TI-RADS classification, providing finer risk stratification. Additionally, few studies have explored SII association with thyroid nodules, and none have integrated TI-RADS and METS-IR for a comprehensive analysis. By combining these novel indices, this study reveals the synergistic effects of insulin resistance and inflammation across nodule grades. The large sample size (n = 49,835) enhances statistical power and generalizability.

Clinically, METS-IR and SII hold significant potential. Both can be calculated from routine blood tests, making them cost-effective and practical. Incorporating these indices into health checkups could identify high-risk individuals, particularly those prone to higher-grade nodules, enabling early intervention and personalized management. For instance, patients with elevated METS-IR or SII could undergo more frequent ultrasound monitoring. Interventions targeting insulin resistance and immune inflammation, such as dietary improvements and increased physical activity, may reduce nodule risk, offering preventive strategies.

The etiology of thyroid nodules is complex and influenced by multiple factors. Recent studies indicate a significant positive correlation between poor adherence to the Mediterranean diet and differentiated thyroid carcinoma (28). It may also be associated with gut microbiota dysbiosis in thyroid nodules, characterized by markedly reduced intestinal butyrate production and butyrate-promoting bacterial populations (29). This study has limitations. Its single-center cross-sectional design may introduce regional bias, necessitating multicenter validation. This design also cannot establish a causal relationship between METS-IR, SII, and thyroid nodules. Other potential confounding factors (such as genetic background, radiation exposure, smoking status, iodine intake, or dietary habits) were not considered. METS-IR and SII may act as mediating factors for various variables, thereby promoting the development and progression of thyroid nodules. Finally, the lack of long-term follow-up data limits our understanding of how changes in METS-IR and SII influence nodule progression.

In conclusion, this study reveals that female gender and older age are major risk factors for thyroid nodules, while low TSH levels correlate with relatively low-grade thyroid nodules, and high METS-IR and SII levels correlate with relatively high-grade thyroid nodules. These findings underscore the role of insulin resistance and systemic inflammation in the pathophysiology of thyroid nodules and suggest that METS-IR and SII may serve as potential biomarkers for monitoring high-risk populations during health screenings. Further research is needed to validate their clinical utility and explore their role in the prevention and treatment of thyroid and metabolic diseases.

## Data Availability

The raw data supporting the conclusions of this article will be made available by the authors, without undue reservation.
